# Identification and characterisation of lamprey protein kinase C delta-like gene

**DOI:** 10.1038/s41598-017-12526-w

**Published:** 2017-09-22

**Authors:** Yang Xu, Siwei Zhu, Huan Zhao, Qingwei Li

**Affiliations:** 1 College of Life Science, Liaoning Normal University, Dalian, 116081 China; 2 Lamprey Research Center, Liaoning Normal University, Dalian, 116081 China

## Abstract

Protein kinase C-δ (PKC-δ), a member of the lipid-regulated serine/threonine PKC family, has been implicated in a wide range of important cellular processes, such as cell growth, differentiation, and apoptosis. Lampreys belong to the most primitive class of vertebrates, and there is little information on PKC-δ in these animals. In this study, a PKC-δ-like cDNA sequence and deduced PKC-δ-like amino acid sequence were identified in the Japanese lamprey (*Lampetra japonica*). The PKC-δ-like gene shared approximately 60% sequence identity with its homologs in jawed vertebrates. The anti-PKC-δ-like polyclonal antibodies were well prepared, and experiments showed that PKC-δ-like was primarily distributed in the supraneural body of the lamprey. Both mRNA and protein levels of PKC-δ-like in supraneural body cells were increased after incubation with cis-diaminedichloroplatinum (CDDP). Moreover, PKC-δ-like protein induced the apoptosis of HEK-293T cells. In addition, the activation of PKC-δ-like resulted in apoptosis. Conversely, the inhibition of PKC-δ-like activity disrupted the CDDP-mediated induction of cellular apoptosis. These results indicate that PKC-δ-like identified in lampreys might play an important role in apoptosis in jawless vertebrates.

## Introduction

Protein kinase C (PKC), a family of phospholipid-dependent serine/threonine protein kinases, comprises a multigene family of related serine/threonine kinases that sit at the crossroads of many signal transduction pathways and regulate a wide variety of cellular functions, including cell proliferation, differentiation and cell death. According to their domain structures and regulation mechanisms, the 12 isoforms in the PKC family are classified into three subgroups: the classical PKCs (α, β1, β2 and γ) that are activated by diacyl glycerol (DAG) and calcium, the novel PKCs (δ, ε, η and θ) that are activated by DAG, and the atypical PKCs (ζ and λ/ι) that respond to neither DAG nor calcium^1–4^. Protein kinase C-δ (PKC-δ) was cloned from a rat cDNA library in 1987^5^. This kinase is a ubiquitously expressed isoform that is ontogenetically regulated in various tissues, such as the brain and epidermis. PKC-δ is the first identified and most thoroughly studied member of the novel PKC subfamily^6^. PKC-δ plays an important role in many cellular signalling pathways, such as cell growth, differentiation, and apoptosis, particularly in the apoptotic pathway^7–10^. Studies have shown that human PKC-δ promotes cell apoptosis, and abnormalities in apoptosis are the main causes of cancer^11–13^. In a variety of human cancers, such as breast cancer, pancreatic cancer, colorectal cancer and liver cancer, the gene mutation or abnormal expression of PKC-δ has been observed^14–16^.

Lampreys (*Lampetra japonicum*) belong to the superclass Cyclostomata and represent the most ancient group of vertebrates^17^. Existing for more than 360 million years, lampreys are considered living fossils due to their many evolutionarily conserved features. From the perspective of genetic information, the lamprey genome remains primitive compared with those of higher vertebrates^18,19^. Lampreys have a unique immune system, as these animals use variable lymphocyte receptors (VLRs) as counterparts of the immunoglobulin-based receptors in jawed vertebrates to specifically recognise and respond to external pathogens^20–23^. In the adult lamprey, there is a special type of fatty tissue rod embedded in the fibro-cartilaginous sheath dorsal to the nerve cord, which was confirmed as the principal haematopoietic organ^24^. This tissue is termed the supraneural body. Studies have shown that the supraneural body is not only associated with haematopoiesis but is also involved in lamprey immunity because numerous immune molecules have been identified in this organ^25–27^. Because of their unique position at the interface between jawless and jawed vertebrates, lampreys are considered one of the most important research models for understanding the origin and evolution of vertebrates^28,29^.

In contrast to the extensive studies on PKC-δ in jawed vertebrates, little is known about the biological activities and physiological roles of PKC-δ in jawless lampreys, and none of the PKC family members in lamprey have been previously identified. Here, we report the cloning of the full-length PKC-δ-like gene in *Lampetra japonica* (*L*. *japonica*) and confirm the tissue distribution and intracellular localization of PKC-δ-like in *L*. *japonica*. In addition, the potential role of PKC-δ-like in the apoptosis of lamprey cells was studied.

## Results

### Cloning, amino acid sequence analysis of PKC-δ-like cDNA

**Figure 1:**
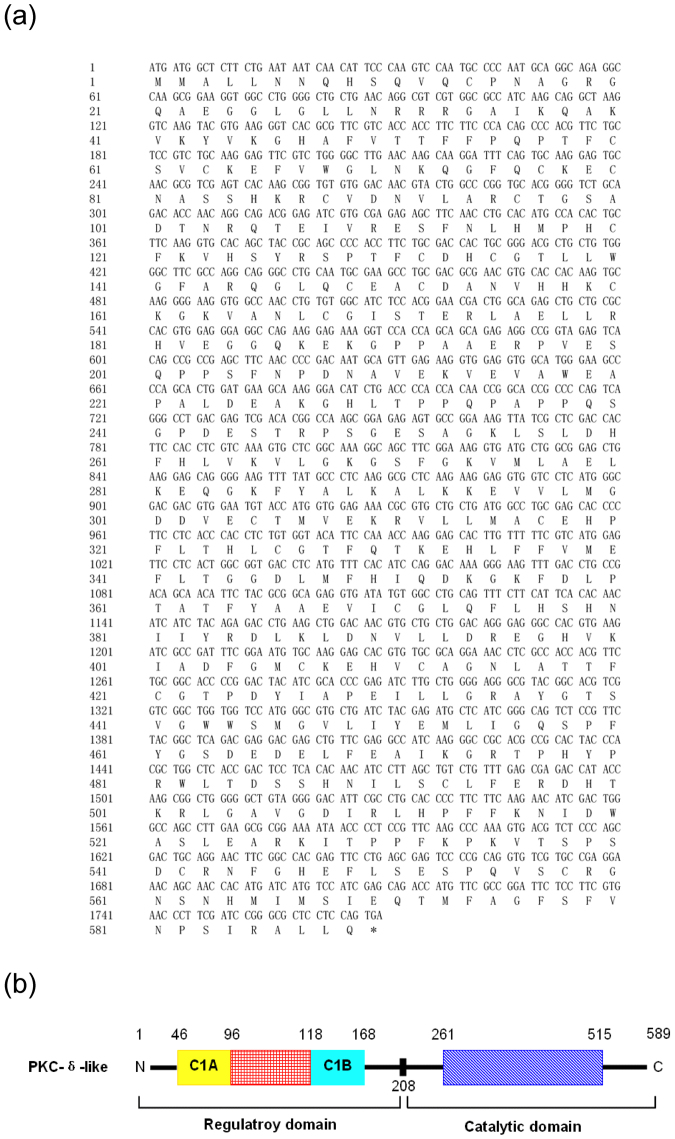
Sequence analysis and the predicted domains of PKC-δ-like from *L*. *japonica*. (**a**) The open reading frame (ORF) of PKC-δ-like. The stop codon is indicated with an asterisk. (**b**) Prediction of the domains of PKC-δ-like.

A single expressed sequence tags (EST) sequence homologous to human PKC-δ was identified among the extensive EST sequences obtained from the cDNA library of *L*. *japonica* supraneural body. PCR analysis, followed by 3′-RACE and 5′-RACE, revealed a full-length PKC-δ-like cDNA sequence of 3298 nucleotides, which contained a 1770-bp open reading frame (ORF) encoding 589 amino acid (aa) residues, a 101-bp 5′-untranslated region (UTR) and a 1427-bp 3′-UTR. The PKC-δ-like cDNA sequence has been submitted to GenBank under the accession number KX943554. Figure 1a shows the complete coding sequences and deduced amino acid sequences of PKC-δ-like. Subsequently, we performed a BLASTp analysis of the full-length protein sequence of PKC-δ-like, and all of the significant alignments were with PKC-δ homologs of different species with high identities (https://blast.ncbi.nlm.nih.gov/Blast.cgi). This outcome confirmed that this gene was homologous to PKC-δ. Thus, henceforth, we refer to this gene as “PKC-δ-like”. The theoretical isoelectric point (pI) of PKC-δ-like protein is 7.54 (http://us.expasy.org/tools/). The PKC-δ-like protein has no signal peptide, indicating that this protein is not secreted (http://www.cbs.dtu.dk/services/SignalP/), and no transmembrane domain, suggesting that PKC-δ-like is not a transmembrane protein (http://www.cbs.dtu.dk/services/TMHMM/).

### Multiple sequence alignment and phylogenetic analysis of the PKC-δ-like protein

**Figure 2:**
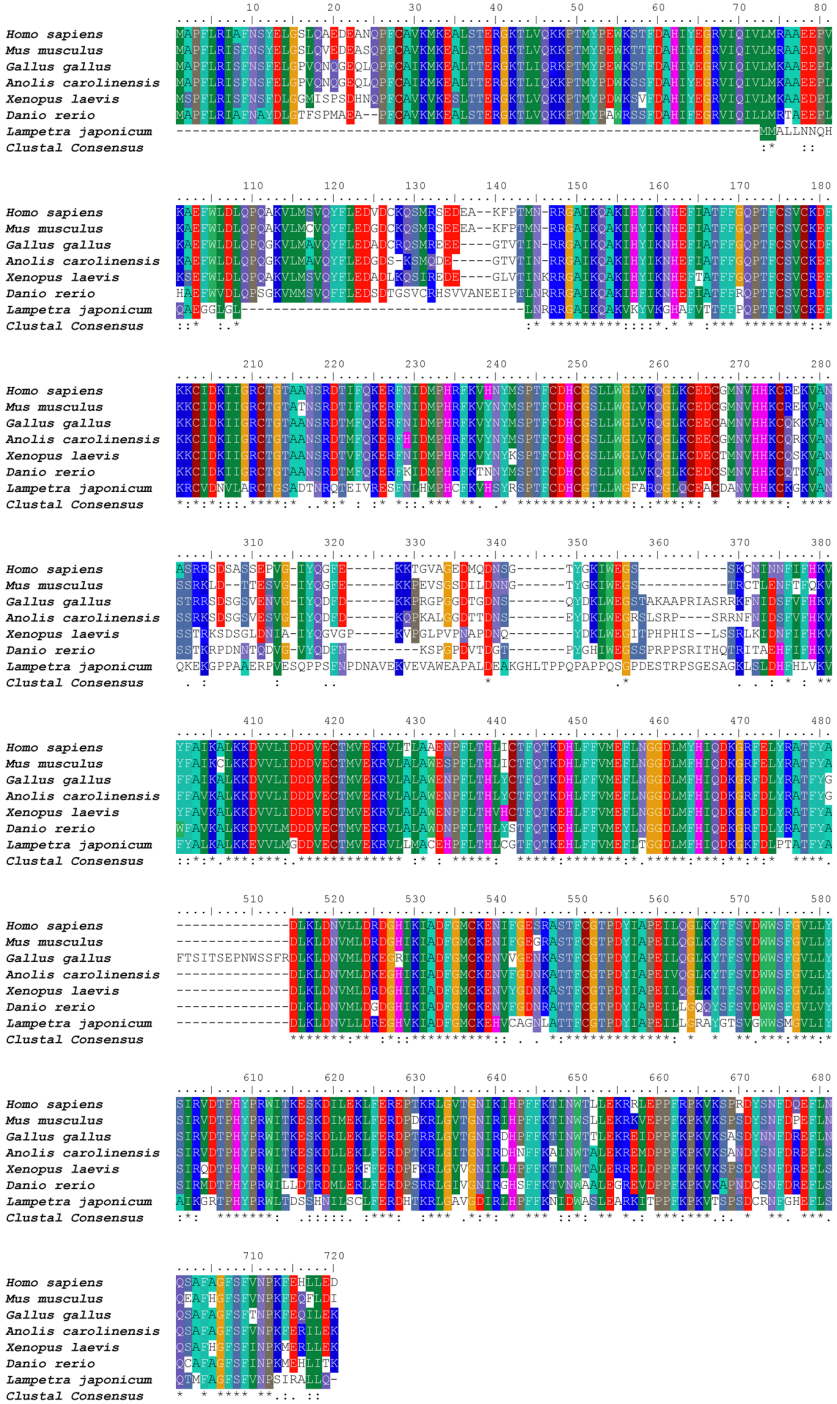
Multiple sequence alignment of PKC-δ-like with PKC-δs from other species. The following accession numbers of the amino acid sequences were extracted from the NCBI protein database: *Homo sapiens*: NP_006245; *Mus musculus*: NP_035233; *Gallus gallus*: NP_001006133; *Anolis carolinensis*: XP_003217703; *Xenopus laevis*: NP_001084460; *Danio rerio*: NP_999873; and *Lampetra japonicum*: KX943554.

**Figure 3:**
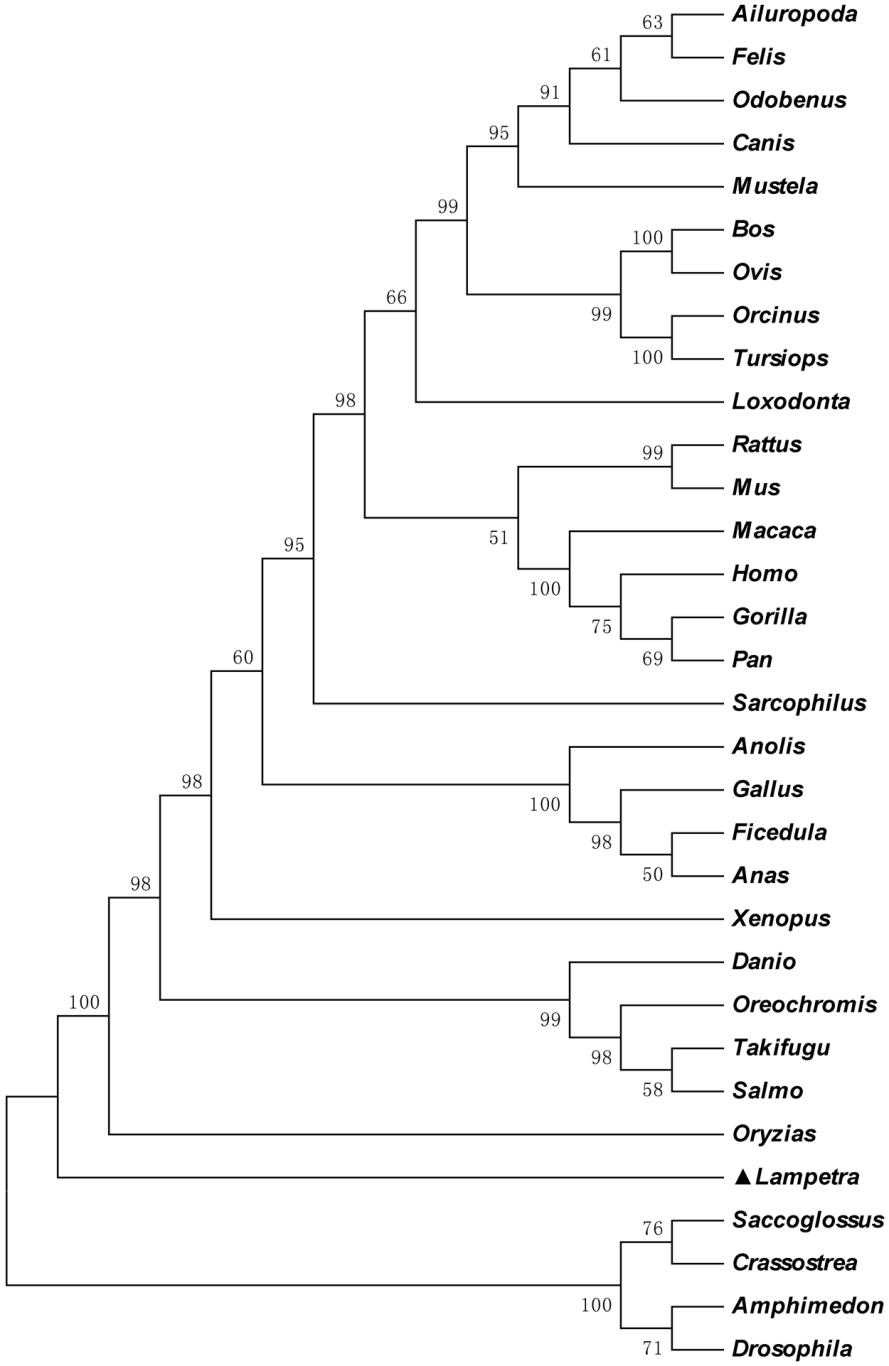
A neighbour-joining phylogenetic tree analysis of PKC-δ protein. The phylogenetic tree was constructed using the neighbour-joining method. The following PKC-δ accession numbers for each species were used: *Homo sapiens*: NP_006245; *Gorilla*: XP_018879336; *Pan troglodytes*: XP_016796770; *Macaca mulatta*: XP_005547448; *Ailuropoda melanoleuca*: XP_002919033; *Loxodonta africana*: XP_010588045; *Bos taurus*: NP_001071323; *Ovis aries*: XP_004018435; *Canis lupus familiaris*: XP_005632396; *Felis catus*: XP_003982374; *Rattus norvegicus*: CAB75578; *Mus musculus*: EDL24758; *Mustela putorius furo*: XP_012908345; *Sarcophilus harrisii*: XP_012399202; *Odobenus rosmarus divergens*: XP_004411513; *Orcinus orca*: XP_004267962; *Tursiops truncates*: XP_004312145; *Gallus gallus*: NP_001006133; *Ficedula albicollis*: XP_005053013; *Anas platyrhynchos*: EOA97230; *Anolis carolinensis*: XP_003217703; *Xenopus laevis*: NP_001084460; *Salmo salar*: XP_014022605; *Oryzias latipes*: XP_011473358; *Danio rerio*: NP_999873; *Oreochromis niloticus*: XP_003441509; *Takifugu rubripes*: XP_003973226; *Lampetra japonicum*: KX943554; *Saccoglossus kowalevskii*: XP_002740313; *Drosophila melanogaster*: NP_511171; *Amphimedon queenslandica*: XP_003384983; and *Crassostrea gigas*: XP_011435403.

ExPAsy website online analysis revealed the structure domain of PKC-δ-like (Fig. 1b). PKC-δ-like has a regulatory domain (1–208 aa) and a catalytic domain (209–589 aa). The regulatory domain contains two cysteine-rich domains: C1A (46–96 aa) and C1B (118–168 aa). The protein kinase domain is located from amino acid 261 to 515, and the active site is 385D. A GenBank database search was conducted to select PKC-δ sequences from *Homo sapiens*, *Mus musculus*, *Gallus gallus*, *Anolis carolinensis*, *Xenopus laevis*, and *Danio rerio*. The multiple sequence alignments revealed that PKC-δ-like possessed 58%, 59%, 58%, 59%, 60% and 60% homology with PKC-δs from humans, mice, birds, reptiles, amphibians, and fishes, respectively (Fig. 2). To examine the evolutionary relationship of *L*. *japonica* PKC-δ-like in jawed vertebrates and invertebrates, a phylogenetic analysis was conducted using the neighbour-joining (NJ) method. A phylogenetic tree was reconstructed with 32 homologs identified from invertebrates to mammals (Fig. 3). The topology of the resulting NJ tree (Fig. 3) revealed that PKC-δ-like of lampreys should be placed between the PKC-δs of invertebrates and those of vertebrates in accordance with the evolutionary position of lampreys.

### Purification of PKC-δ-like recombinant protein and preparation of polyclonal antibodies

**Figure 4:**
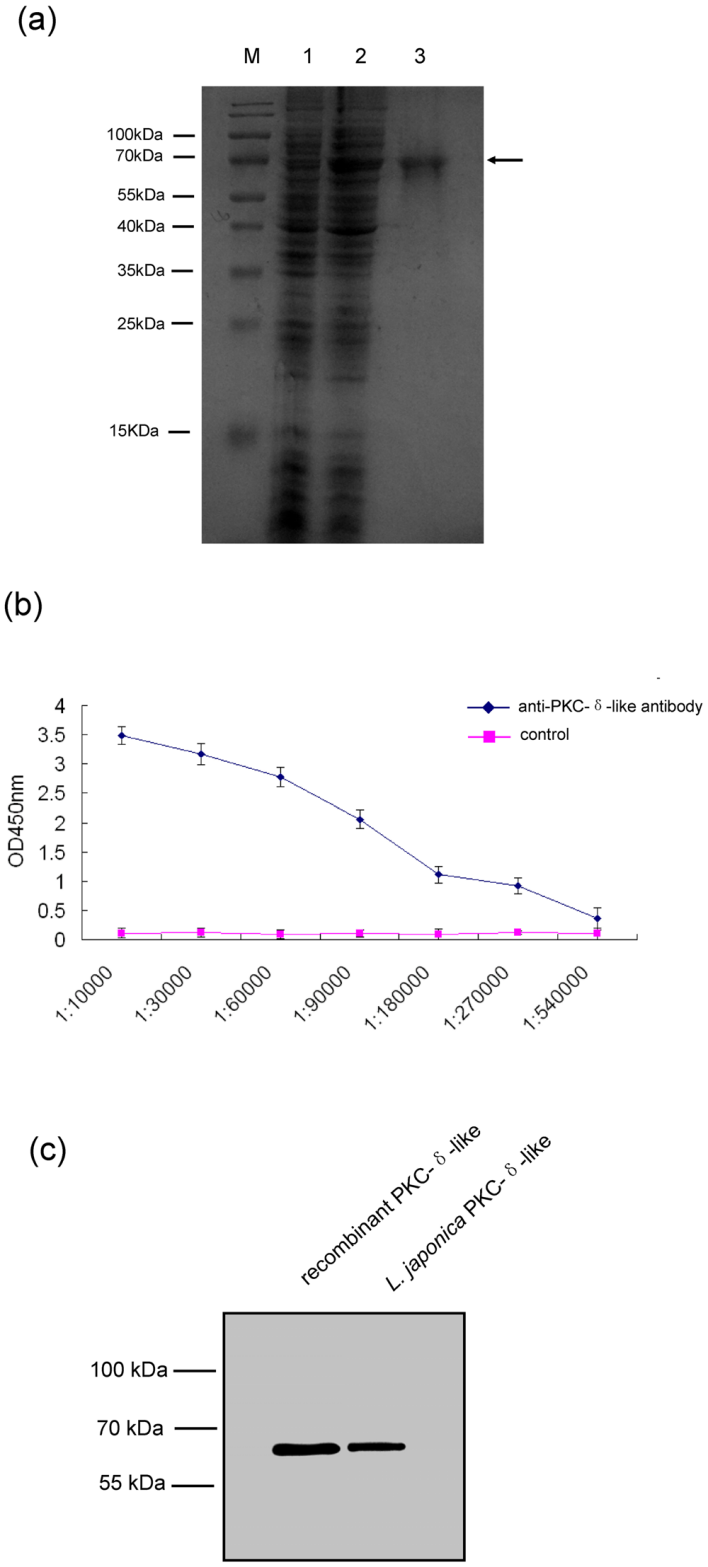
Purification of recombinant PKC-δ-like protein and preparation of polyclonal antibodies. (**a**) Expression and purification of *L*. *japonica* PKC-δ-like recombinant protein. The PKC-δ-like recombinant protein was expressed in *E*. *coli* BL21 and analyzed through SDS-PAGE. M, protein marker; lane 1, total protein of uninduced cells; lane 2, total protein of induced cells containing pET-28a-PKC-δ-like; lane 3, purified PKC-δ-like recombinant protein. (**b**) Indirect ELISA of PKC-δ-like IgG with PKC-δ-like recombinant protein. Serially diluted (1:10,000–1:540,000) polyclonal antibodies were tested against PKC-δ-like recombinant protein using ELISA. Pre-immune IgG was used as a negative control (n = 3). Error bars indicate the standard error of the mean. (**c**) Western blot analysis of the specificity of rabbit anti-PKC-δ-like polyclonal antibodies. Lane 1, PKC-δ-like recombinant protein; lane 2, supraneural body from *L*. *japonica*. Full-length blots are presented in Supplementary Fig. S1.

**Figure 5:**
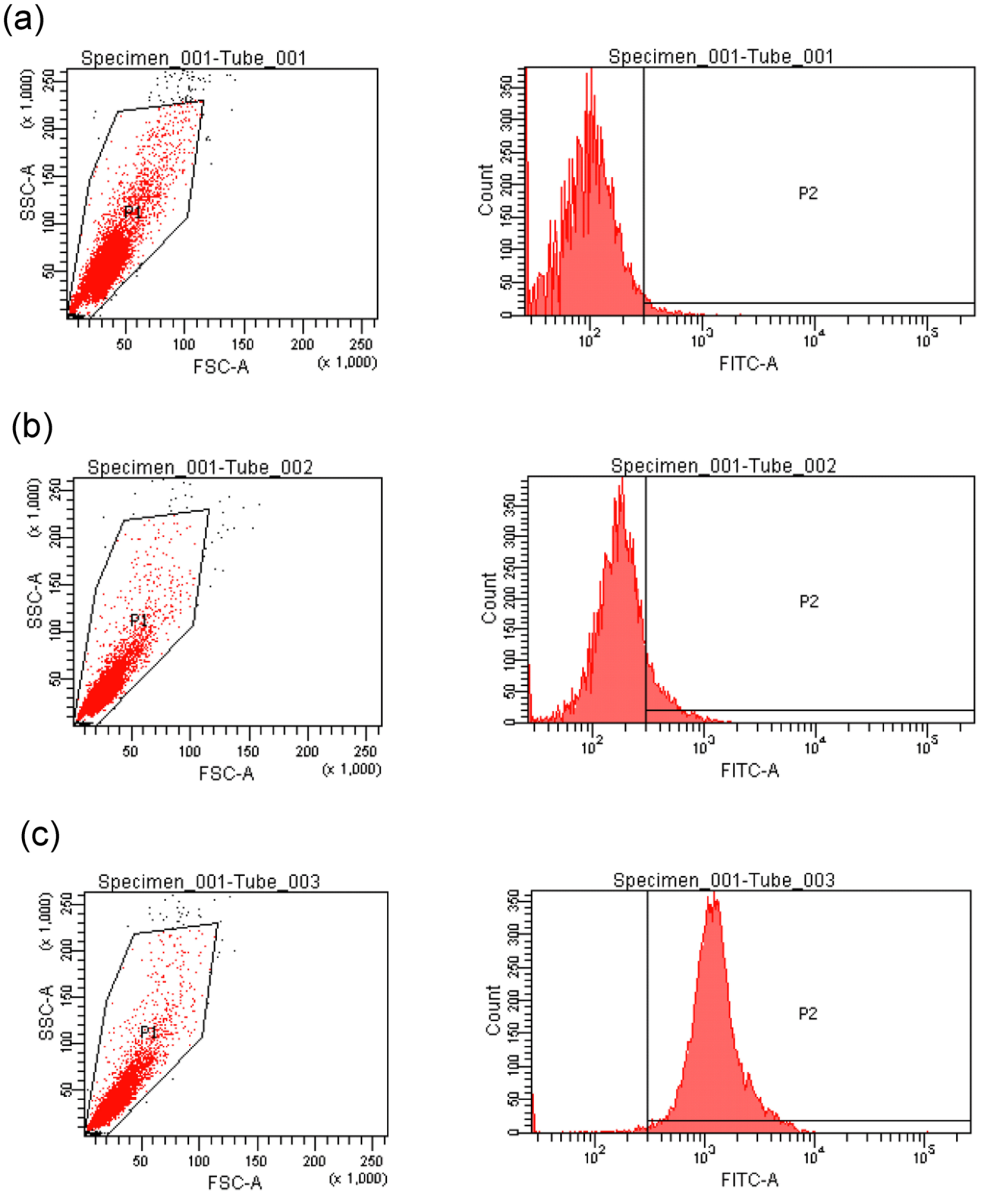
FACS analysis of PKC-δ-like in supraneural body cells. Supraneural body cells, bound to the anti-PKC-δ-like polyclonal antibodies, were incubated with FITC-conjugated anti-rabbit IgG secondary antibodies followed by FACS analysis. The cells were initially selected using light scattering (FSC, forward scatter; SSC, side scatter). The anti-PKC-δ-like polyclonal antibodies and an FITC-conjugated secondary antibody were added to the supraneural body cells. (**a**) The supraneural body cells were incubated without antibodies. (**b**) Supraneural body cells bound to polyclonal antibodies from pre-immunized rabbit serum prior to incubating with FITC-conjugated secondary antibodies were used as controls. (**c**) Supraneural body cells bound to anti-PKC-δ-like polyclonal antibodies were incubated with FITC-conjugated anti-rabbit IgG secondary antibodies.

To express recombinant PKC-δ-like, the PKC-δ-like gene was cloned into the vector pET-28a. After induction using isopropyl-1-thio-b-D-galactopyranoside (IPTG), PKC-δ-like was expressed as a histidine-tagged fusion protein in *Escherichia coli* BL21 (DE3). The purified recombinant PKC-δ-like protein migrated as a single band on a 10% SDS-PAGE gel with a molecular mass of approximately 70 kDa, consistent with the molecular mass predicted from the DNA sequence (Fig. 4a). The polyclonal antiserum was successfully prepared as described in the Methods section. The titres of the obtained antibodies were analysed using enzyme linked immunosorbent assay (ELISA). The results showed that the titre of the rabbit anti-PKC-δ-like polyclonal antibodies was higher than 1: 270,000 (Fig. 4b) using pre-immunized rabbit immunoglobulin G (IgG) as a negative control. The specificity of the antibody and expression of *L*. *japonica* PKC-δ-like in the supraneural body were determined through western blot analysis. The antibody detected both the recombinant PKC-δ-like protein and the native PKC-δ-like protein in the supraneural body (Fig. 4c). To further evaluate the expression of PKC-δ-like in the supraneural body, these cells were incubated with anti-PKC-δ-like protein polyclonal antibodies and analyzed via flow cytometry (Fig. 5). Fluorescence-activated cell sorting (FACS) analysis revealed that PKC-δ-like was expressed in supraneural body cells, consistent with the western blotting results.

### Expression of the PKC-δ-like in adult tissues and its localization in cells

**Figure 6:**
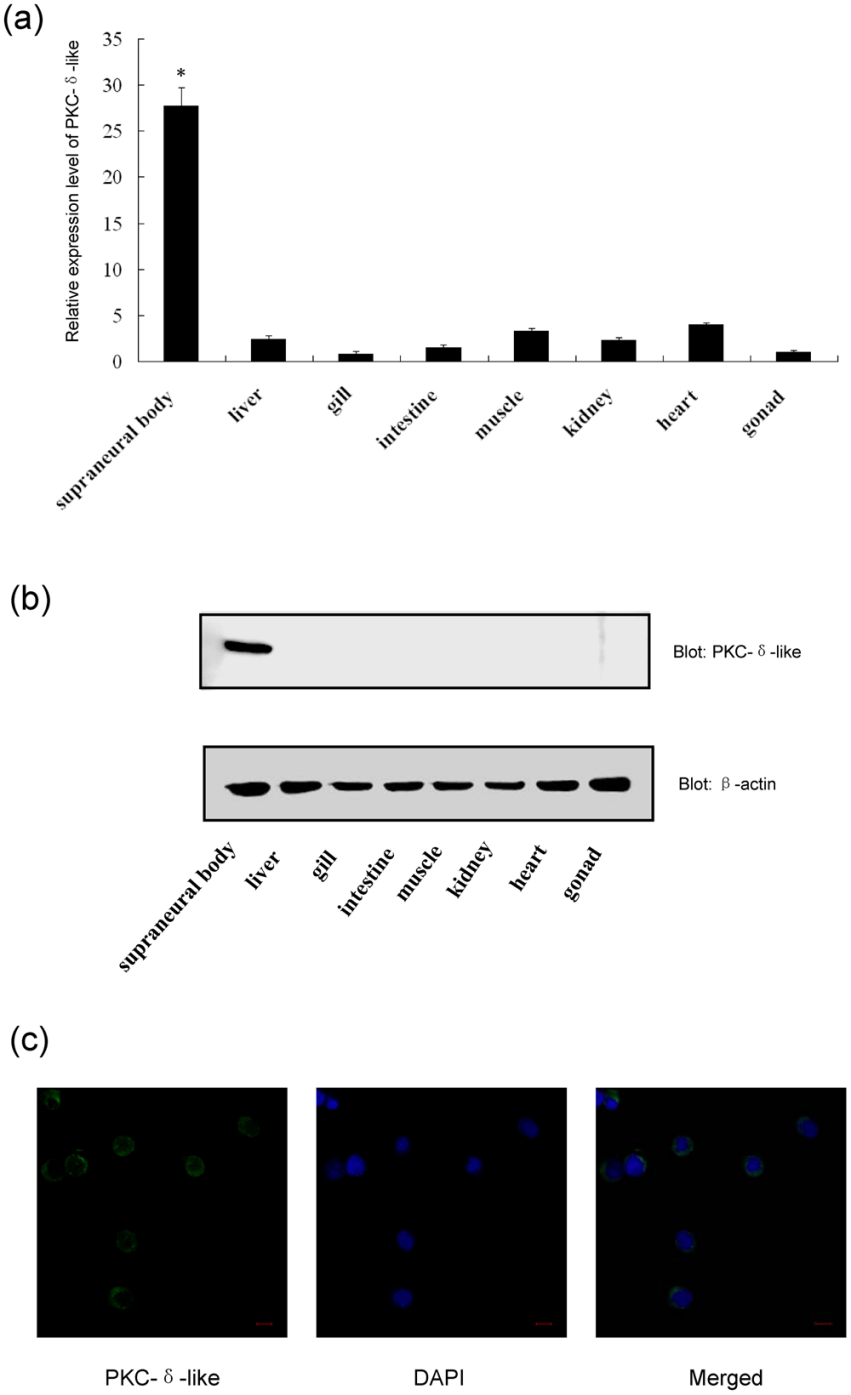
Expression of PKC-δ-like in adult tissues and localization in cells. (**a**) The expression of PKC-δ-like was measured using real-time PCR. The mRNA level of PKC-δ-like is expressed as fold-change relative to the GAPDH mRNA level. The relative expression of PKC-δ-like mRNA in tissues versus the gonads was calculated using 2^−ΔΔCT^, in which ΔCt = Ct(PKC-δ-like) − Ct(GAPDH), ΔΔCT = ΔCt(various tissue) − ΔCt(gonad). All data were calculated from three parallel experiments and are shown as the means ± SD. *P < 0.05. (**b**) Western blotting analysis of the PKC-δ-like levels in *L*. *japonica* tissues. Proteins from crude homogenates of various lamprey tissues were probed with a rabbit anti-PKC-δ-like antibody. Lanes 1–8: supraneural body (lane 1), liver (lane 2), gill (lane 3), intestine (lane 4), muscle (lane 5), kidney(lane 6), heart (lane 7) and gonad (line 8) from *L*. *japonica*. (**c**) Immunofluorescence detection of PKC-δ-like in supraneural body cells. The primary antibody was the anti-PKC-δ-like antibody (100-fold), and the secondary antibody was an FITC-conjugated goat anti-rabbit IgG antibody (200-fold). The cells were stained with DAPI. The immunofluorescence was visualized and captured using a fluorescence microscope (Carl Zeiss, Inc.). Scale bar: 5 μm. Full-length blots are presented in Supplementary Fig. S1.

The expression of PKC-δ-like in *L*. *japonica* tissues was analyzed using real-time PCR (Fig. 6a). In all instances, the GAPDH gene was successfully amplified, indicating the validity of the cDNA template. Tissue expression profiling revealed the constitutive expression of PKC-δ-like in all the tissues examined, including the supraneural body, liver, gill, intestine, muscle, kidney, heart and gonad. The highest expression was detected in the supraneural body (28-fold relative to the PKC-δ-like mRNA level in the gonad). However, PKC-δ-like could be detected only in the supraneural body through western blotting (Fig. 6b). To investigate the intracellular localization of PKC-δ-like, the distribution of PKC-δ-like in *L*. *japonica* supraneural body cells was further detected using immunofluorescence and confocal laser-scanning microscopy, which demonstrated that PKC-δ-like protein is localized in both the cytoplasm and nucleus of these cells (Fig. 6c).

### Impact of apoptosis on the transcription and expression of PKC-δ-like

**Figure 7:**
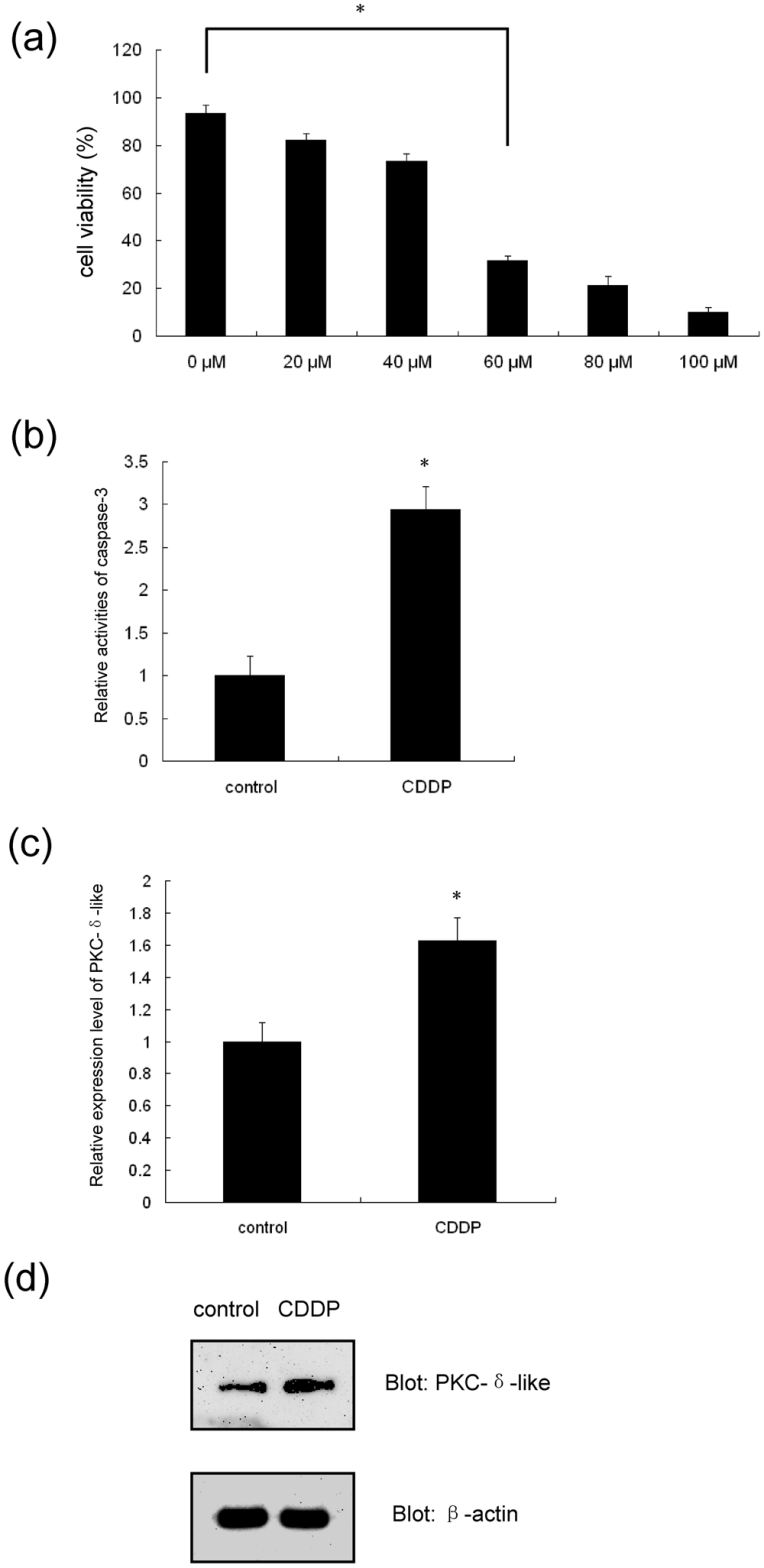
The effect of apoptosis on the expression of PKC-δ-like. (**a**) Concentration-dependent change in cell viability. The supraneural body cells were treated with CDDP for 6 h at 0 μM, 20 μM, 40 μM, 60 μM, 80 μM and 100 μM, separately. The viability of the cells was detected using trypan blue staining. (**b**) Examination of caspase-3 activity in supraneural body cells with or without CDDP treatment. (**c**) Change of PKC-δ-like mRNA levels in supraneural body cells with or without CDDP treatment. (**d**) Detection of PKC-δ-like protein levels in supraneural body cells with or without CDDP treatment. All data were calculated from three parallel experiments and are shown as the means ± SD. *P < 0.05. Full-length blots are presented in Supplementary Fig. S1.

The cells were separated from the supraneural body and subsequently treated with CDDP at different concentrations for 6 h. CDDP, also termed cisplatin, is a chemotherapeutic drug used to treat a number of cancers^30,31^. Here, CDDP was used to induce apoptosis. The viability of supraneural body cells was detected using trypan blue staining (Fig. 7a). Because more than half of the cells died after treatment with 60 μM CDDP, we used a concentration between 40 and 60 μM (50 μM CDDP) to induce apoptosis. Subsequently, the caspase-3 activity in the cells was detected. The results showed that the caspase-3 activity in CDDP-treated cells was enhanced by approximately 3-fold compared with the negative control (Fig. 7b). These data indicated that CDDP induced cell apoptosis. To explore whether the expression level of PKC-δ-like changed during apoptosis, real-time PCR and western blotting were performed. The results shown in Fig. 7c reveal that the transcriptional level of PKC-δ-like increased in apoptosis, and the results shown in Fig. 7d show that the protein expression level of PKC-δ-like increased after CDDP treatment.

### Effect of the expression and activity of PKC-δ-like on apoptosis

**Figure 8:**
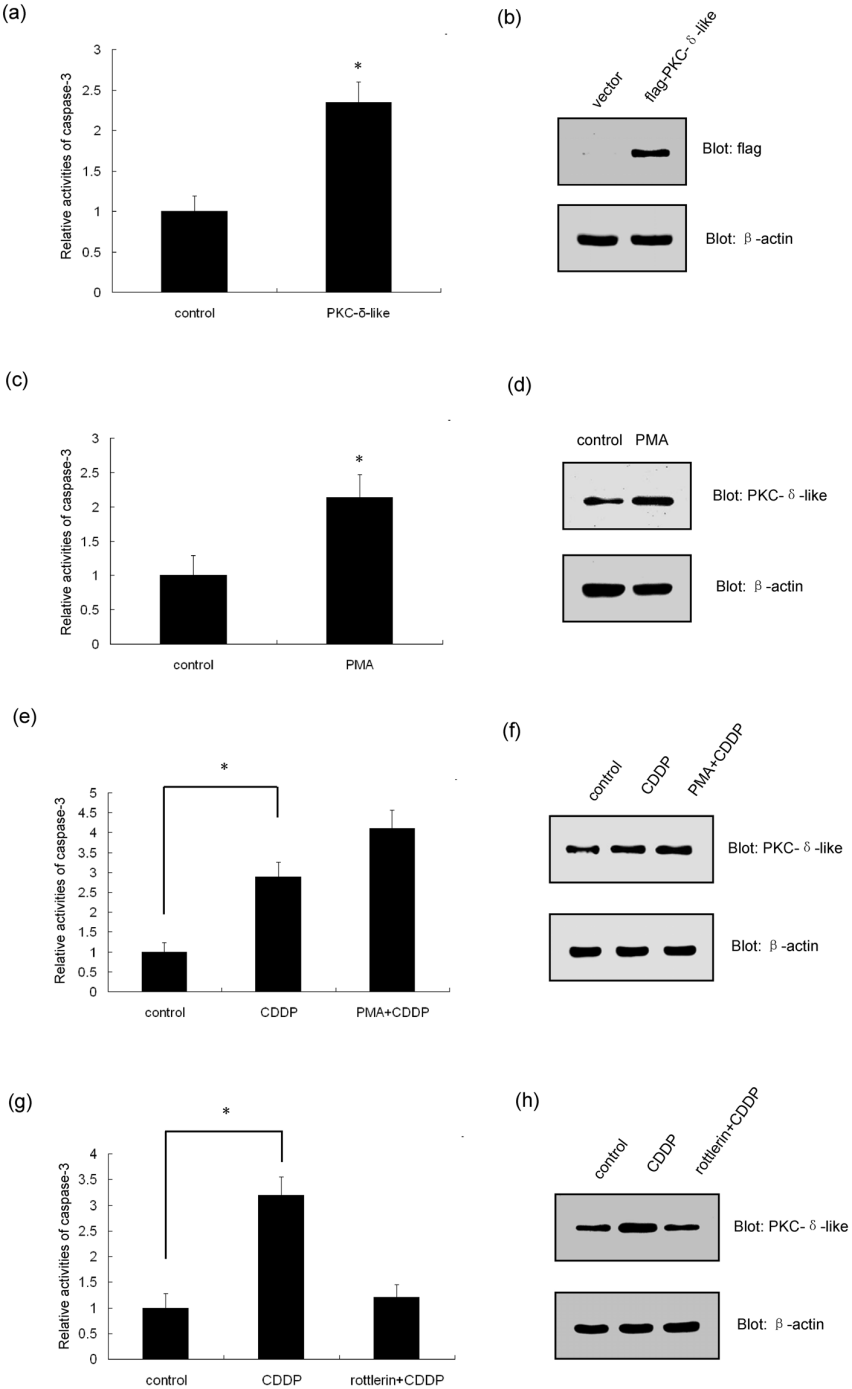
The effect of PKC-δ-like expression and activity on apoptosis. (**a**) Examination of caspase-3 activity in HEK-293T cells transfected with pcDNA3.1-flag (control) or pcDNA3.1-flag-PKC-δ-like. (**b**) Detection of PKC-δ-like protein levels in HEK-293T cells transfected with pcDNA3.1-flag (control) or pcDNA3.1-flag-PKC-δ-like. (**c**) Examination of caspase-3 activity in supraneural body cells with or without PMA treatment. (**d**) Detection of PKC-δ-like protein levels in supraneural body cells with or without PMA treatment. (**e**) Examination of caspase-3 activity in supraneural body cells treated with control or CDDP or PMA + CDDP. (**f**) Detection of PKC-δ-like protein levels in supraneural body cells treated with control or CDDP or PMA + CDDP. (**g**) Examination of caspase-3 activity in supraneural body cells treated with control or CDDP or rottlerin + CDDP. (**h**) Detection of PKC-δ-like protein levels in supraneural body cells treated with control or CDDP or rottlerin + CDDP. All data were calculated from three parallel experiments and are shown as the means ± SD. *P < 0.05. Full-length blots are presented in Supplementary Fig. S1.

To further investigate the role of PKC-δ-like in apoptosis, we over-expressed PKC-δ-like in HEK-293T cells and examined whether PKC-δ-like protein could induce apoptosis. The data showed that increasing PKC-δ-like protein expression triggered apoptosis (Fig. 8a and b). In addition, we investigated whether the change in PKC-δ-like activity could affect apoptosis. Phorbol-12-myristate-13-acetate (PMA) is an activator of PKC-δ^32^. Here, we incubated the supraneural body cells with 200 nmol/L PMA for 9 h. The results showed that PMA can induce apoptosis (Fig. 8c). Moreover, the protein level of PKC-δ-like slightly increased after incubation with PMA (Fig. 8d). Meanwhile, we incubated the supraneural body cells with both 200 nmol/L PMA for 9 h and 50 μM CDDP for 6 h. Figure 8e showed that PMA could enhance CDDP-induced apoptosis. But PMA did not help CDDP to increase the expression of PKC-δ-like evidently (Fig. 8f). In contrast, when supraneural body cells were treated with both 10 μmol/L of rottlerin, an inhibitor of PKC-δ^33^, and 50 μM CDDP for 6 h, rottlerin effectively prevented CDDP from inducing apoptosis (Fig. 8g) and inhibited the increase of PKC-δ-like protein induced after CDDP treatment (Fig. 8h).

## Discussion

Lamprey research was initiated as early as the 19^th^ century^34^. With the emergence of modern molecular techniques, lampreys have become an ideal animal model for studying vertebrate evolution, embryo development, and the origin of some cellular signalling pathways^17,19,29^. However, due to the incomplete genome information for lamprey, the sequences of many important genes in lampreys remain unknown, making it difficult to study the cellular signalling pathways of these animals. Therefore, obtaining the full-length sequences of crucial genes and determining their functions is of great value.

Apoptosis, a genetically determined cell suicidal programme that removes unwanted, redundant and damaged cells, is required to maintain the balance between cell proliferation and cell death. A defect in apoptotic machinery can lead to cancer^35–38^. Although PKC is a class of protein kinases that have been discovered early, some controversies concerning the role and molecular mechanism of different PKC subtypes in apoptosis remain. In general, human PKC-δ has been implicated in promoting apoptosis, while human PKC-α has been implicated in the inhibition of apoptosis^39–41^. The different roles of different PKC subtypes in apoptosis increase the difficulties and obstacles encountered in selecting the correct targets for cancer therapies. However, a recent article published in “*Cell*” changed the original view. A total of 554 types of PKC mutants and their activities were analyzed in human tumours. The results showed that most PKC subtypes (such as PKC-α) inhibit tumour formation and promote apoptosis, which is not consistent with the results of previous studies^42^. The results of the present study indicated that there is still much to uncover about the role of PKC-δ in apoptosis. As lampreys occupy a critical phylogenetic position in understanding the origin and evolution of the apoptosis signalling pathway, it is important to identify the PKC-δ homolog in lamprey.

Here, we present the first isolation and characterisation of the full-length PKC-δ-like cDNA from *L*. *japonica*. The BLASTp results illustrated that this gene should be homologous to PKC-δ. Sequence analysis data implied that PKC-δ-like should have similar kinase catalytic activity to that of human PKC-δ. The phylogenetic analysis showed that the evolutionary status of PKC-δ-like gene was relatively primitive between amphioxus and teleost fish, consistent with the law of evolution from lower animals to higher animals.

To further analyse the potential involvement of PKC-δ-like in apoptosis in the lamprey, we generated anti-PKC-δ-like polyclonal antibodies with high specificity. The distribution of PKC-δ-like in different tissues of lamprey showed an interesting phenomenon. Unlike human PKC-δ, we detected PKC-δ-like protein in the supraneural body. As noted above, the supraneural body is an important immune organ in lamprey^25^. The high expression level of PKC-δ-like in the supraneural body indicated that this protein might play a role in lamprey immunity, although the relevance of the function of PKC-δ-like in apoptosis and its role in immunity should be explored in future studies. We validated that more PKC-δ-like is distributed in the cytoplasm and nucleus of supraneural body cells, and more PKC-δ-like protein was found in the cytoplasm than in the nucleus (Fig. 6c). This phenomenon was consistent with human PKC-δ, likely indicating that most PKC-δ-like protein remains in the cytoplasm, and some activated PKC-δ-like proteins will translocate to the nucleus to fulfil kinase functions^11,43^.

Since human PKC-δ plays a crucial role in cell apoptosis, we determined whether PKC-δ-like was also involved in the apoptosis of lamprey cells. First, we investigated the relationship between the expression of PKC-δ-like and apoptosis. It was shown that apoptosis increased the PKC-δ-like expression levels, and the over-expression of PKC-δ-like protein also induced apoptosis. These data indicated a positive correlation between apoptosis and the protein level of PKC-δ-like. Thus, PKC-δ-like may play a positive role in apoptosis in the lamprey. Next, we determined whether the activity of PKC-δ-like was also involved in apoptosis. The activation of PKC-δ-like could trigger apoptosis (Fig. 8c). Conversely, the inhibition of PKC-δ-like activity dramatically attenuated CDDP-induced apoptosis (Fig. 8g). Meanwhile, PMA and CDDP had a synergistic effect on apoptosis. These results indicated that CDDP-induced apoptosis in lamprey was mainly dependent on the expression and activation of PKC-δ-like. Thus, this reflected the crucial role of PKC-δ-like in CDDP-induced apoptosis. PKC-δ-like should be one kind of kinases according to its homology with human PKC-δ. Many core proteins of the pathways participated in CDDP-induced apoptosis are the substrates of PKC-δ in human, such as p53, DNA-dependent protein kinase (DNA-PK), c-abl and so on^44–46^. Therefore, we speculate that PKC-δ-like in lamprey should also be located in the upstream of the cell signalling pathways associated with CDDP-induced apoptosis. Briefly, the function of PKC-δ-like in lamprey apoptosis is conserved compared with human PKC-δ. Notably, both of these proteins promote apoptosis. However, the precise role of PKC-δ-like in apoptosis and the specific substrates of PKC-δ-like are unclear. Thus, further studies are needed to shed more light on this field and increase the current understanding of the mechanism through which the PKC-δ-like protein fulfils its function as a crucial molecule in apoptosis in lampreys.

## Methods

### Animals

Fresh male adult *L*. *japonica* (lengths: 36.4~58.4 cm, weights: 112~274.5 g) were obtained from the Tongjiang Valley of Songhua River, Heilongjiang Province, China in December, 2015. This work was approved by the Animal Welfare and Research Ethics Committee of the Institute of Dalian Medical University (Permit Number: SYXK2004–0029), and the methods were performed in accordance with the approved guidelines.

### Cloning of PKC-δ-like cDNA from *L*. *japonica*

Through analyzing the expressed sequence tags (EST) of the cDNA library constructed with supraneural body of *L*. *japonica* by our lab previously, a PKC-δ homologue (PKC-δ-like) was found using Basic Local Alignment Search Tool (BLAST) in the National Center for Biotechnology Information (NCBI). Total RNAs from supraneural body cells of *L*. *japonica* were extracted based on RNAiso (TaKaRa Biotechnology, Dalian, China) reagent and then converted to cDNA by reverse transcription using PrimeScript^TM^ II first Strand cDNA Synthesis Kit (TaKaRa Biotechnology, Dalian, China). Full-length cDNA was amplified using the 3′-RACE Core Set Kit (TaKaRa Biotechnology, Dalian, China) and 5′-RACE SMARTer^TM^ RACE cDNA Amplification Kit (Clontech Laboratories, USA) with the 3′-RACE primer and 5′-RACE primer listed in Table 1. All PCR products were analyzed by electrophoresis in a 1% agarose gel stained with ethidium bromide. The target band of PCR product was isolated and purified, subcloned into a pMD19-T vector using a DNA Ligation Kit (TaKaRa Biotechnology, Dalian, China), and transformed into DH5α *Escherichia coli*. The recombinant plasmid was then isolated and subjected to DNA sequencing (TaKaRa Biotechnology, Dalian, China).

**Table 1:** Oligonucleotide primers used in the study.

Primer	Sequence (5′-3′)
RACE	
3′-RACE-Outer	AGATGCTCATCGGGCAGTCTCCGTTCTACG
3′-RACE-Inner	GCAACATCCTCAGCTGTCTGTTTGAGC
5′-RACE-Outer	AAATCCTTGCTTGTTCAAGCCCCAGACGAA
5′-RACE-Inner	GACGAACGCGTGACCCTTCACGAACT
Realtime-PCR	
PKC-δ-like-forward	GCATCTCCACGGAACGAC
PKC-δ-like-reverse	CCACCTCCACCTTCTCAACT
GAPDH-forward	ACCCCTTCATTGACCTGGAGTA
GAPDH-reverse	TGCTTACCCCATGGGATGTT

### Amino acid sequence analysis and phylogenetic analysis

The amino acid sequence deduced from the full-length cDNA of PKC-δ-like was analyzed by online tool at http://www.expasy.org/tools/scanprosite. The domain of PKC-δ-like was predicted on ExPAsy (http://prosite.expasy.org/). Multiple sequence alignments were performed using ClustalX (http://www.ebi.ac.uk/Tools/clustalw/) and BioEdit. A phylogenetic tree was constructed using the MEGA 4 program by the neighbor-joining (NJ) or maximum parsimony methods based on the pair-wise deletion of gaps/missing data and a p-distance matrix of an amino acid model with 1000 bootstrap replicates.

### Expression and purification of PKC-δ-like recombinant protein

A 1767-bp fragment of the open reading frame (ORF) of PKC-δ-like cDNA (GenBank: KX943554) encoding 589 amino acids flanked by a BamH I and a Xho I restriction site, was amplified and subcloned into the pET-28a expression vector. The recombinant protein was expressed in *Escherichia coli* BL21 (DE3) by induction with 0.1 mM isopropyl-1-thio-b-D-galactopyranoside (IPTG) for 3.5 h. Subsequently, the cells were harvested by centrifugation, washed, and resuspended in banding buffer (20 mM Tris-HCl, pH 8.0, 50 mM NaCl, and 20 mM imidazole). The cells in banding buffer were lysed by sonicating for 30 min on ice and centrifuged again at 16,000 × g for 10 min at 4 °C. The soluble supernatant was collected and purified with Ni affinity chromatography (GE Healthcare, New York, NY, USA). The concentration of PKC-δ-like recombinant protein determined by the Bradford method and the purity of the sample was analyzed by 10% SDS-PAGE.

### Production of polyclonal antibodies

Polyclonal antibodies against the PKC-δ-like recombinant protein were raised in male New Zealand white rabbits. Each animal was immunized for four consecutive injections with a gap of 2 weeks per immunization. For the first immunization, 400 μg of purified PKC-δ-like recombinant protein in 500 μL PBS was incorporated and emulsified with an equal volume of Freund’s complete adjuvant (Sigma-Aldrich St. Louis, MO, USA). For the subsequent three immunizations, 200 μg of the protein in 500 μL PBS was incorporated and emulsified with an equal volume of Freund’s incomplete adjuvant (Sigma-Aldrich St. Louis, MO, USA). Peripheral blood was centrifuged at 7100 × g for 5 min and the antiserum was collected. Then polyclonal antibodies (anti-PKC-δ-like) were purified from antiserum by protein G affinity chromatography (GE Healthcare, New York, NY, USA). The concentration of purified anti-PKC-δ-like was adjusted to 1 mg/mL and stored at −20 °C in 50% glycerol. The antibody titer was determined by enzyme-linked immunosorbent assay (ELISA). The specificity of the antibodies was confirmed by western blot assay using the PKC-δ-like recombinant protein and the lysates of supraneural bodys from *L*. *japonica*.

### Real-time PCR

The expression of PKC-δ-like messenger RNA (mRNA) was examined by real-time quantitative PCR detecting system (qPCR). Total RNAs were separated from lamprey tissues including supraneural body, liver, gill, intestine, muscle, kidney, heart and gonad using RNAiso reagent (TaKaRa Biotechnology, Dalian, China). The total RNAs were treated with DNase I (TaKaRa Biotechnology, Dalian, China) and then subjected to reverse transcription using PrimeScript™ RT Reagent Kit (Perfect Real Time) (TaKaRa Biotechnology, Dalian, China). The qPCR experiments were performed with a TaKaRa TP800 Real Time PCR System (TaKaRa Biotechnology, Dalian, China) using 2 μL cDNA with 16.8 μL SYBR green PCR mastermix (TaKaRa Biotechnology, Dalian, China) and 0.6 μL of each specific primer (Table 1). The glyceraldehyde-3-phosphatedehydrogenase (GAPDH) of lamprey was used as an internal control to normalize the starting quantity of RNA. Results were expressed as the mean ± SD of three biological parallel experiments for each specimen.

### Western blotting

The tissue or cells were lysed in lysis buffer containing 1% 3-[(3-cholamidopropyl) dimethylammonio]-1-propanesulphonate (CHAPS) (SigmaeAldrich St. Louis, MO, USA), protease inhibitor cocktail (Roche, Mannheim, Germany) and phenylmethanesulphonyl fluoride (PMSF) (SigmaeAldrich St. Louis, MO, USA). The lysates were centrifuged for 20 min at 13,000 × g at 4 °C and then 20 μL supernatant was added to 20 μL of 2 × loading buffer and boiled at 100 °C for 5 min. The proteins were analyzed by SDS-PAGE under reduced conditions, transferred to a polyvinylidene difluoride (PVDF) membrane (Millipore Corporation, Billerica, MA, USA) and blocked with 5% fat-free milk in PBS plus 0.05% Tween (PBS-T). After being washed three times in PBS-T, the membrane was incubated with anti-PKC-δ-like antibody (0.5 μg/mL) or anti-flag antibody (Cell Signaling Technology, MA, USA) (0.5 μg/mL) in PBS-T and 5% fat-free milk for 1 h at 37 °C. After washing, the membrane was incubated with horseradish peroxidase (HRP)-conjugated goat anti-rabbit IgG in PBS-T and 5% fat-free milk for 30 min at 37 °C. The signals were revealed using enhanced chemiluminescence (ECL) kit (Pierce, Rockford, IL, USA). Rabbit anti-β-Actin (Zhongshanjinqiao, Beijing, China) was used to normalize the amount of protein per lane.

The western blot images were captured by using FluorChem Q (ProteinSimple, CA, USA). The PVDF membrane was incubated with ECL for 1 minute, and then it was put on the chemiluminescence plate of the machine. The aperture factor is adjusted to 0.95. Set the filter to “1”. Then open the software of this machine, choose “Auto Expose” and click “Acquire” to capture the western blot images. Save the pictures as tiff or jpeg files.

### Cell isolation

The lampreys were dissected and then wiped with 70% alcohol. The supraneural bodys were stripped from the lampreys. After washing with ice-cold phosphate-buffered saline (PBS) and saturated with RPMI-1640 media supplemented with antibiotics (100 U/mL of penicillin sulfate and 100 μg/mL of streptomycin), the supraneural bodys were cut into small pieces approximately 1 × 1 mm^2^ with scissors and transferred to 25 cm^2^ cell culture flasks containing 30 mL 0.25% trypsin solution. The culture flasks were maintained at 4 °C for 12 h. The next day, the cells released into the solution were decanted, centrifuged at 350 × g for 5 min, and transferred to 1640 medium supplemented with 100 U/mL of penicillin sulfate and 100 μg/mL of streptomycin at 4 °C for 3 days. Then, the cells were separated by centrifugation. To remove dead cells, Lymphocyte Separation Kit (Solarbio, Beijing, China) was used to obtain the living cells.

### Fluorescence-activated cell sorting (FACS) analysis

*L*. *japonica* supraneural body cells were plated in tubes and fixed for 20 min in 90% methanol in PBS at room temperature. Then, the cells were washed three times with PBS, blocked with normal goat serum for 30 min, and incubated with rabbit anti-PKC-δ-like (200-fold) in PBS for 1 h at room temperature. After being washed three times, the cells were incubated with FITC-conjugated donkey anti-rabbit IgG (500-fold) for 45 min at room temperature in the dark followed by three washes with PBS. The cells were resuspended with PBS and analyzed on a FACS Aria flow cytometer (BD Biosciences). Cells incubated with FITC-conjugated goat anti-rabbit IgG were used as isotype controls. Data analysis was performed using Flowjo software (Tree Star).

### Immunofluorescence

*L*. *japonica* supraneural body cells were plated in tubes and fixed for 25 min in 4% paraformaldehyde in PBS at room temperature. Cells were washed twice with PBS. The cells were fixed and permeabilized for 10 min using 0.1% Triton X-100, blocked with normal goat serum for 30 min, and incubated with primary antibody rabbit anti-PKC-δ-like (100-fold) at 4 °C overnight. After the overnight incubation, the cells were washed twice with PBS and then incubated with FITC-conjugated goat anti-rabbit IgG antibody (200-fold). Following two more washes with PBS, the cells were stained with DAPI (200-fold). After two washes with PBS, the coverslips were mounted on glass slides with one drop of antifade solution. The immunofluorescence was visualized and captured with a Zeiss LSM 780 inverted microscope (Carl Zeiss, Inc) and analyzed using Zeiss ZEN LE software.

### Assessment of cell viability by trypan blue staining

Trypan blue is a vital stain which is used to selectively color dead cells blue, while leaving live cells with intact cell membranes not colored. Briefly, cell suspension was diluted with equal volume (1: 1) of trypan blue and left for 5 min and then loaded to the hemocytometer slide. Cells were counted under microscope as the bright cells were considered viable while the blue ones were considered dead. The total number of viable cells was calculated using the following equations:

$${\rm{The}}\,{\rm{number}}\,{\rm{of}}\,{\rm{viable}}\,{\rm{cells}}\,{\rm{per}}\,{\rm{milliliter}}={\rm{Average}}\,{\rm{number}}\,{\rm{of}}\,{\rm{viable}}\,{\rm{cells}}\times {\rm{dilution}}\,{\rm{multiple}}\times {10}^{4}$$

### Caspase-3 activity assay

Caspase-3 activity in the cells was analyzed by means of Caspase-3 Colorimetric Assay kit (KeyGen, Nanjing, China) following the manufacturer’s instructions. In brief, cells were lysed in ice bath for 1 h and vortexed every 20 min. Then the tissue lysates were centrifuged for 10 min at 12,000 × g at 4 °C. The supernatant were diluted to 50 μL using cell lysis buffer, incubated with 5 μL of substrate at 37 °C for 4 h in dark and a microplate reader (SpectraMax i3x Multi-mode detection platform; Molecular Devices Inc) was used to determine the absorbance of the samples at 405 nm to quantify the caspase-3 activity.

### Cell culture and transfection

HEK-293T cells were cultured in DMEM supplemented with 10% fetal bovine serum (FBS) in a 5% CO_2_ incubator at 37 °C, overnight. Then, cells were transfected with pcDNA3.1-flag or pcDNA3.1-flag-PKC-δ-like. The transfection of the plasmids into the cells was performed using lipofectamine 2000 (Thermo Fisher Scientific, MA, USA) according to the manufacturer’s instructions.

### Statistical analysis

Data were presented as mean ± SD of at least three experiments. The differences between different groups were analyzed using student’s *t* test and analysis of variance (ANOVA). *P* < 0.05 was considered statistically significant.

### Data availability statement

The datasets generated during and/or analysed during the current study are available from the corresponding author on reasonable request.

## Electronic supplementary material

Supplementary information
