# Supplementary material for: Identification and characterisation of lamprey protein kinase C delta-like gene
Source: Sci Rep. 2017 Sep 22;7:12214. doi: 10.1038/s41598-017-12526-w (PMC5610172; doi:10.1038/s41598-017-12526-w)

# Identification and characterisation of lamprey protein kinase C delta-like gene

Yang Xu[1,2,*], Siwei Zhu[1,2,*], Huan Zhao[1,2] & Qingwei Li[1,2]

[1]College of Life Science, Liaoning Normal University, Dalian 116081, China.

[2]Lamprey Research Center, Liaoning Normal University, Dalian 116081, China.

[*]These authors contributed equally to this work.

Correspondence and requests for materials should be addressed to Q.L. (email: liqingwei2020@163.com)

## Supplementary Information

**Supplementary Fig. S1.** Full-length western blots. (a) Full-length gels of Fig. 4 c. (b and c) Full-length gels of Fig. 6 b. (d and e) Full-length gels of Fig. 7 d. (f and g) Full-length gels of Fig. 8 b. (h and i) Full-length gels of Fig. 8 d. (j and k) Full-length gels of Fig. 8 f. (l and m) Full-length gels of Fig. 8 h.

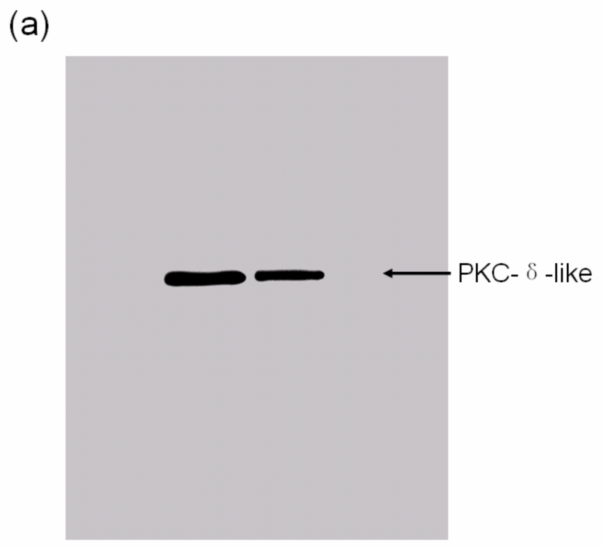


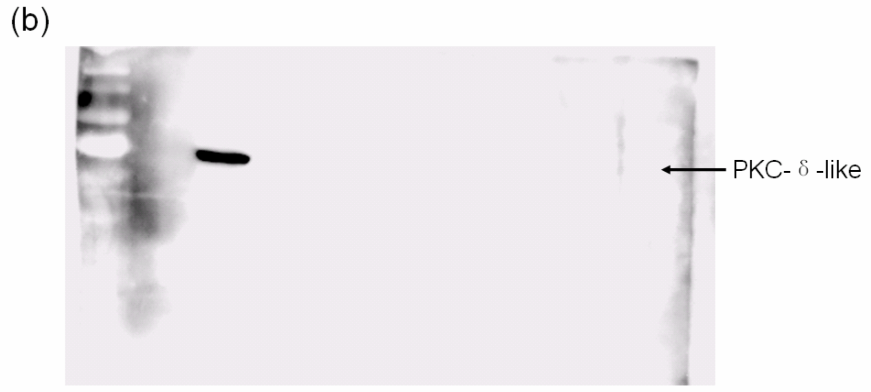

(c)

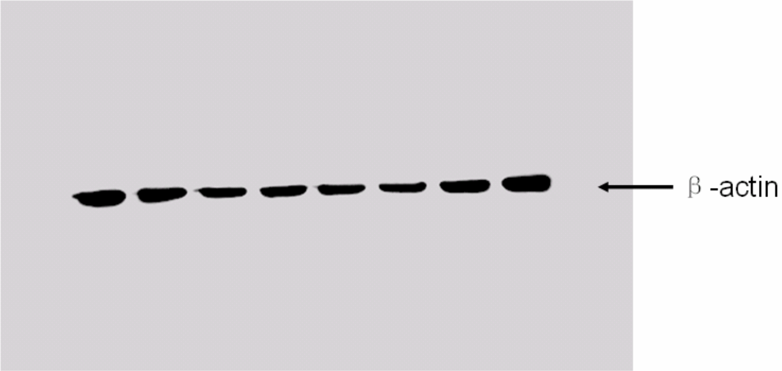


(d)

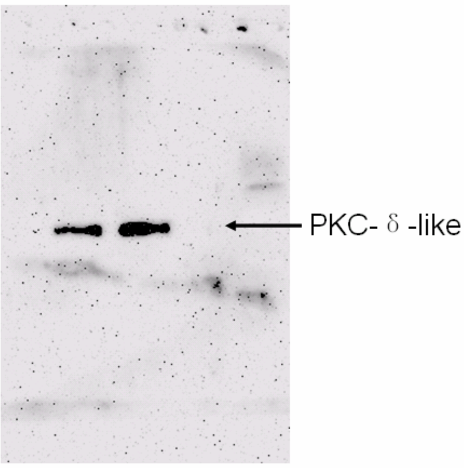


(e)

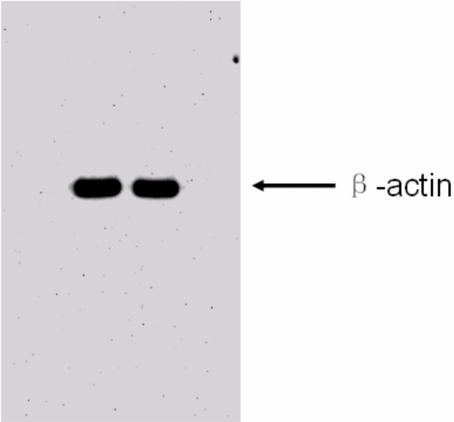

(f)

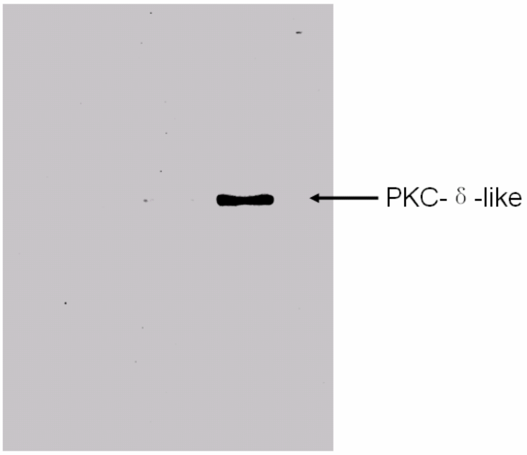


(g)

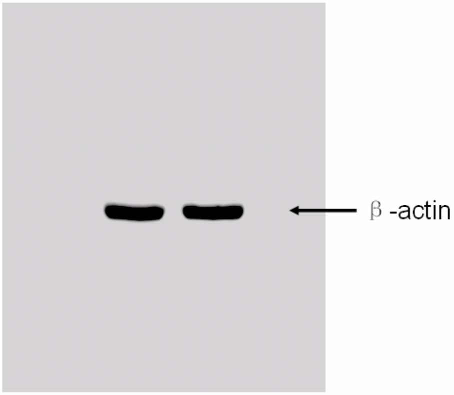


(h)

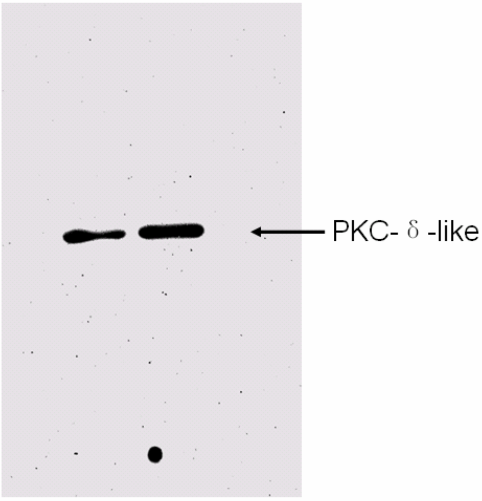

(i)

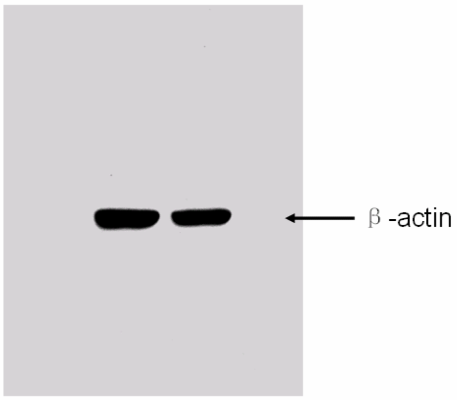


(j)

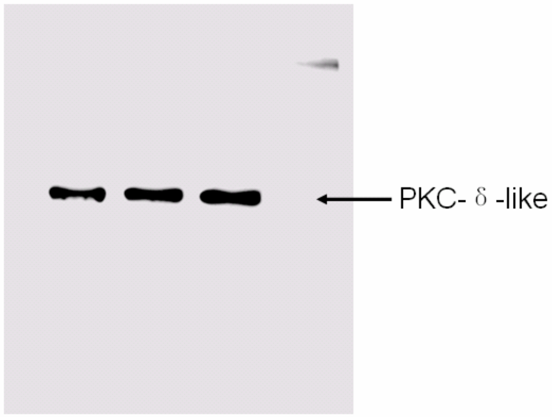


(k)

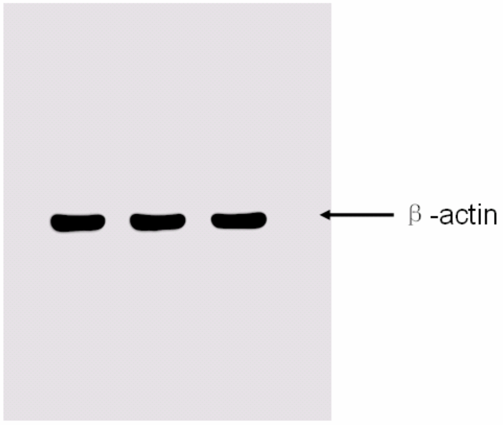

(l)

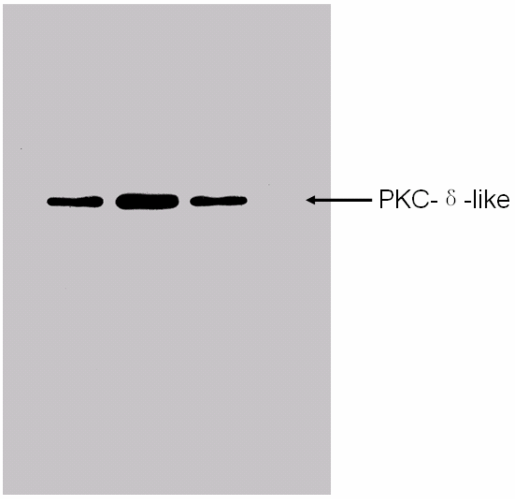


(m)

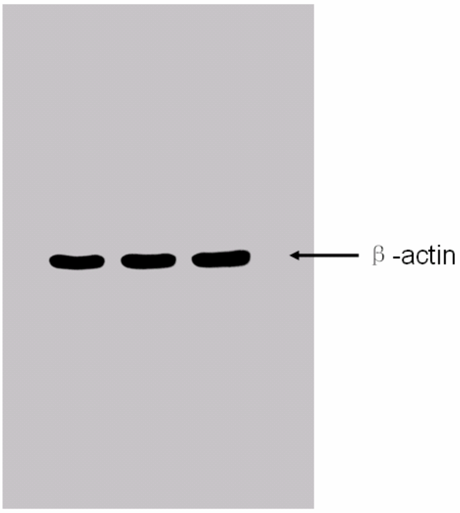

Supplement: Supplementary file 1 — Supplementary information [file 41598_2017_12526_MOESM1_ESM.pdf]
